# Association Between Suicide-Related Outcomes and Primary Care Outpatient Visits: Impact of Mental Health Screening and Interventions

**DOI:** 10.7759/cureus.86356

**Published:** 2025-06-19

**Authors:** Afolake A Adebayo, Chekwube M Obianyo, Kemi Johnson, Jennifer C Mbonu, Toluwalase Erukusin, Violet C Mokwenye, Opemipo Adetifa, Omotola Akinade, Okelue E Okobi, Abisola R Adeyemi

**Affiliations:** 1 Department of Family Medicine, Nnamdi Azikiwe University, Nnewi, NGA; 2 Jiann-Ping Hsu College of Public Health, Georgia Southern University, Statesboro, USA; 3 Department of Clinical Psychology, Walden University, Minneapolis, USA; 4 Department of Social Work/Medicine and Surgery, Hennepin Healthcare, Minneapolis, USA; 5 Department of Social Work/Medicine and Surgery, Crimean Federal University, Simferopol, RUS; 6 Department of General Medicine, Usmanu Danfodiyo University, Sokoto, Sokoto, NGA; 7 Department of General Practice, University of Uyo, Uyo, NGA; 8 Department of Psychiatry, Internal Medicine, Surgery, Obstetrics, and Gynecology, National Hospital Abuja, Abuja, NGA; 9 Department of Family Medicine, Kyiv Medical University, Kyiv, UKR; 10 Department of Internal Medicine, General Hospital Ikorodu, Lagos, NGA; 11 Department of Family Medicine, Larkin Community Hospital Palm Springs Campus, Miami, USA; 12 Department of Family Medicine, Lakeside Medical Center, Belle Glade, USA; 13 Department of Psychiatry, Alberta Health Services - Rockyview General Hospital, Calgary, CAN

**Keywords:** mental health service, outpatient clinics, physician-patient relationship, primary healthcare, suicide, suicide screening

## Abstract

Suicide remains a major public health concern, although most individuals who experience suicidal thoughts and ideations have contacted primary care shortly before committing suicide. This systematic review highlights the importance of suicide risk screening and prevention interventions in primary care contexts. Nevertheless, despite the different primary care practices being widely employed in the screening of different mental health concerns, including depression, most individuals feel unprepared or uncomfortable with screening for suicide risk. Therefore, the objective of this study is to systematically examine and synthesize existing evidence on the relationship between suicide-related outcomes (including suicidal ideation, suicide attempts, and suicide deaths) and primary care outpatient visits, with particular emphasis on the impact of mental health screening and intervention practices implemented within these settings.

To attain the objective, a systematic search was conducted in PubMed, Google Scholar, Web of Science, Scopus, and Embase for peer-reviewed studies published between 2010 and 2025. Studies were included if they examined suicide-related outcomes (ideation, attempts, or death) in relation to primary care visits and incorporated data on mental health screening or interventions in these settings. Two reviewers independently screened articles, extracted data, and assessed methodological quality using standardized tools. Due to study heterogeneity, a narrative synthesis was used. Thus, of the 15 studies meeting inclusion criteria, most reported that a significant proportion of individuals who died by suicide had contact with primary care providers within one year before death. However, the implementation of mental health screening or suicide risk assessments during these visits was inconsistent. Studies that incorporated structured screening tools or brief interventions within primary care showed a potential reduction in suicide-related outcomes, although evidence quality varied. Primary care outpatient visits represent a pivotal point for suicide prevention. Enhanced integration of mental health screening and intervention practices in these settings may improve early detection and reduce suicide risk. Further high-quality, longitudinal research is needed to establish effective strategies for routine suicide prevention in primary care.

## Introduction and background

Suicide attempts and related deaths vary by age, sex, race, and other key factors, including prior suicide attempts, childhood trauma, non-suicidal self-harm, and psychiatric disorders [1-5]. Predicting suicidal ideation remains difficult at the individual level. However, the risk of attempts and deaths rises with more developmental, biomedical, and psychosocial stressors, as well as with higher distress levels [6-8].

Luoma et al. reported that 38% of US adults who died by suicide had seen a primary care provider in the month before their death; this rate rises to about 70% among older adults [9]. In the past year, 90% of suicidal youths had at least one primary care visit, compared to 70% of non-suicidal youths [10]. These findings highlight the potential of precise, practical screening tools in primary care to identify high-risk individuals and link them to timely interventions.

Moreover, suicidality, which includes suicide ideation, suicidal thoughts, and self-harm, remains a widespread feature in persons with various kinds of mental disorders [11,12]. According to the World Health Organization, globally, the number of individuals committing suicide annually surpasses 800,000 [13,14]. Of the number, it is estimated that 28% are existing mental health patients [8,10-13] and 66% of individuals who complete suicide are males, 71% are unmarried, and 68% have a history of previous self-harm [11,12].

Suicidal thoughts and behaviors can be distressing for individuals and their families, with significant societal impacts [15]. However, regardless of the higher prevalence rates of suicidality, the existing evidence-based interventions for suicidality remain limited [16,17]. In this regard, psychological interventions have been acknowledged as important in suicidality treatment and prevention, and studies have disclosed that various psychosocial interventions, including cognitive therapy, problem-solving, dialectical behavioral therapy, and outreach interventions, are effective for reducing and preventing suicidality [18,19]. Such interventions have been underpinned by varied theoretical models, with the argument on the similarity of efficiency being pegged on the significance of therapeutic alliance [20]. In this regard, therapeutic alliance has been defined as the confidence of the patients in the healthcare staff and the strength of their existing relationship [21,22].

Suicidal acts have been linked to social and interpersonal challenges, including intricate and traumatic relationships, as well as the lack of vital relationships [22]. The existing relationship between a patient and a healthcare professional is liable to provide the patient with an initially unknown experience of security, an individual who is not only responsive and sensitive but also provides the patient with the much-needed haven [22]. Regardless of the acknowledged significance of developing sturdy therapeutic alliances with individuals who experience suicidality, it is only in recent times that a limited number of empirical studies in this field have been conducted.

Early screening and detection are critical for suicide prevention. Since most individuals who die by suicide see a health provider within a year of their death, medical settings offer prime opportunities for risk identification and referral to lifesaving care [23,24]. Frontline clinicians can engage in practical, adaptable processes for suicide risk screening, evaluation, and management, with support from mental health specialists.

Risk factors for suicide

Among the notable and widespread risk factors for suicide are proximal and distal risk factors. According to Sarkhel et al., proximal risk factors include the increasingly imminent signs such as a sense of hopelessness, major stressful event and loss, suicidal ideation, imprisonment, and access to means [25]. Distal risk factors include the background issues and events such as mental health diagnoses, family history of suicide attempts and completions, and previous suicide attempts [26]. Primary care providers are well-positioned to identify imminent suicide warning signs and address proximal factors. Additionally, Knipe et al. have also linked lower socioeconomic status to increased risk of suicide and suicidal behaviors, even as socioeconomic aspects, including educational disadvantage, deprivation, low income, and poor housing, have been acknowledged to be prevalent in both the proximal and distal risk factors [27]. Normally, individuals who complete suicide have a combination of different proximal and distal suicide risk factors. The probability of death through suicide differs across an array of population demographics, based on aspects such as age, sex, sexuality, occupation, and socioeconomic status [27]. For instance, in most nations, suicide rates have been noted to be higher in males; however, in Asian nations, significant variations have been observed across sexes [28]. Most of the factors have been associated with poor physical health, including chronic diseases, and poor mental health, including mental disorders.

A widespread misconception exists suggesting that suicidality is an issue that is only experienced by individuals undergoing poor mental health. However, it has been disclosed that, of the individuals who complete suicide, only 28% had visited mental health services within 12 months before their deaths [29,30], indicating that not all individuals who die through suicide had poor mental health. Further, tackling aspects of premature deaths and health inequalities is a significant policy driver, even as the prevention of suicides within the at-risk populations is important. Therefore, the objective of this study is to conduct a systematic review and subsequently synthesize extant literature and evidence on the correlations between suicide-linked outcomes and primary care outpatient visits, underlining the impact of mental health screening and intervention practices implemented within primary care contexts.

## Review

Materials and methodology

A comprehensive literature search was conducted across multiple databases, including PubMed, Google Scholar, Web of Science, Scopus, and Embase, for studies published between 2010 and 2025. The selected databases were chosen because together, they cover biomedical and clinical literature (PubMed and Embase), broad multidisciplinary and citation indexing (Web of Science and Scopus), and grey literature and recent conference abstracts (Google Scholar). This combination maximizes comprehensiveness and minimizes publication bias. The selected studies comprised prospective cohort studies, epidemiological studies, health assessment studies, and multicenter studies. Duplicate records were identified and removed by comparing titles, authors, years, and study populations. We used the following Medical Subject Headings (MeSH) terms and keywords:suicide, primary healthcare, physician‑patient relationship, suicide screening, mental health service, and outpatient clinics. The initial search yielded 729 unique articles.

Search Strategy

We conducted our final search on March 1, 2025, using the following Boolean string (example for PubMed): ("suicide"[MeSH Terms] OR "suicid*"[Title/Abstract]) AND ("primary care"[MeSH Terms] OR "general practice"[Title/Abstract] OR "outpatient clinic"[Title/Abstract]) AND ("screening"[Title/Abstract] OR "intervention"[Title/Abstract] OR "risk assessment"[Title/Abstract]). The following filters were used: publication dates from 2010 to 2025 and in the English language. Analogous strings, adapted to each database's syntax, were run in Embase, Scopus, Web of Science, and Google Scholar.

Inclusion and Exclusion Criteria

The removal of identified duplicate studies was followed by the selection of relevant studies, conducted based on a three-phase process. The first phase entailed the screening of the studies' titles and abstracts. The second phase excluded every study found irrelevant to the study. Consequently, the third phase involved a comprehensive full-text evaluation of every study to ascertain its relevance. As such, three independent reviewers were tasked with screening the studies, and potential disagreements were resolved through discussions and consensus.

Further, for this study, the inclusion criteria targeted original studies that included randomized controlled trials (RCTs), prospective cohort studies, and crossover studies that met the following criteria: studies published between 2010 and 2025, original scientific studies published in the English language and peer-reviewed and reputable journals, and full-text articles. To further qualify, the included studies had to focus on assessing the correlation between overweight and obesity and various mental health disorders.

On the other hand, the exclusion criteria were decided a priori by the study team and methodological experts based on the review's objectives and standard systematic review guidance. Criteria were pilot‑tested on a random sample of 50 articles to ensure clarity and reproducibility. We excluded sponsored clinical trials lacking independent data analysis; opinion pieces, narrative reviews, and editorials; studies with irrelevant populations or outcomes; articles without full‑text access; and reports with clearly unsound or unreported methodologies. A total of 700 references were excluded at various stages for these reasons.

Data drawn from included studies included aspects such as general attributes of the studies, author names, and year of publication; demographic attributes, such as sample size, gender, age, follow-ups, and race; and the interventions used, intervention duration, and measurement methods. Three reviewers extracted data independently; disagreements were resolved by consensus. This was followed by the systematic recording of the key study findings.

Results

Study Selection

**Figure 1:**
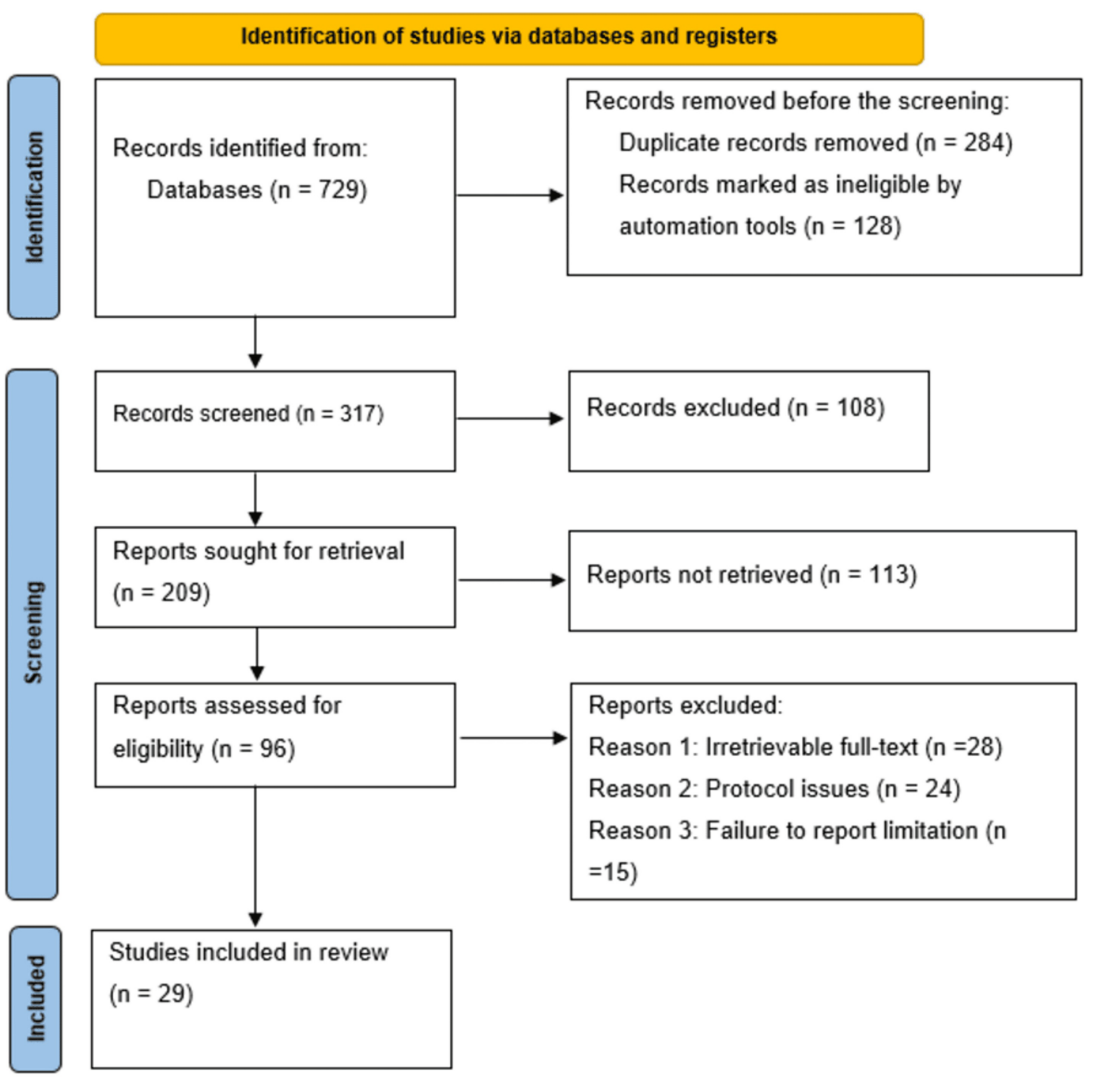
PRISMA flow diagram indicating the study selection process for the included studies PRISMA: Preferred Reporting Items for Systematic Reviews and Meta-Analyses, n: number

For this systematic review, the study selection and inclusion criteria followed the Preferred Reporting Items for Systematic Reviews and Meta-Analyses (PRISMA) guidelines. As a result, the original in-depth database search yielded a total of 729 references. The screening resulted in the exclusion of 284 duplicates alongside 128 references marked as ineligible by automation tools. The subsequent screening of the studies' titles and abstracts resulted in the further exclusion of 108 studies considered ineligible. The researchers sought to retrieve the remaining 209 references and also assessed them for eligibility. Therefore, an additional 113 references were excluded for being irretrievable. As a result, 96 references were assessed for eligibility, and 67 studies were excluded for various reasons, including irretrievable full-texts (28 studies), protocol (24 studies), and failure to sufficiently report the study limitations (15 studies). Eventually, 29 studies satisfied the inclusion criteria and were included in this study, and have also been assessed and discussed together with the findings of other studies that have corroborated this study's findings [31-64]. The PRISMA flow diagram in Figure 1 illustrates the article selection process.

Table 1 provides a summary of the studies included in this review and their findings.

**Table 1:** Summary of the included studies and their findings GP: general practitioner, UK: United Kingdom, PHQ-9: 9-item Patient Health Questionnaire, ED-SAFE: Emergency Department Safety Assessment and Follow-up Evaluation, BITS: bullying, insomnia, tobacco, stress

Authors/citation	Study type	Summary	Findings
Mesec et al. (2010) [31]	Retrospective case-control study	The study assessed the frequency and reasons for primary care visits by the suicide victims in Slovenia.	The study disclosed that 39% of suicide victims visited primary care physicians in the month prior to their deaths, even as 30% of such visits were attributed to mental health concerns, in comparison to 3% in controls.
Schou Pedersen et al. (2019) [32]	Nationwide matched comparative study	The study evaluated healthcare utilization patterns in the year before suicide in Denmark.	The study disclosed that over 30% of individuals who died by suicide had GP contact in the month before death, indicating potential intervention opportunities.
Alothman et al. (2024) [33]	Case-control study	The study assessed primary care consultation patterns before suicide across the UK.	The study disclosed that medication review, depression, and pain were the most widespread reasons underlying the consultations in the year prior to suicide.
Webb et al. (2012) [34]	Case-control study	The study explored suicide risk among primary care patients who had major physical diseases.	The study disclosed that coronary heart disease, stroke, osteoporosis, and chronic obstructive pulmonary disease were associated with increased suicide risk, normally mediated by depression.
Younes et al. (2013) [36]	Cross-sectional study	Investigated challenges faced by GPs with young adults who attempted suicide.	GPs experienced significant difficulties resulting from infrequent consultations and reduced detection of depression in younger adults.
Muñoz-Sánchez et al. (2018) [37]	Qualitative study	The study analyzed the perspectives of healthcare professionals with regard to the management of suicide risk in Spain.	The study identified key factors for suicide prevention across different healthcare settings, highlighting the need for coordinated care.
Richards et al. (2019) [38]	Qualitative study	The study explored patient experiences regarding being asked about suicidality during their regular primary care visits.	Patients valued being asked about suicidality, although some experienced fear of stigma; empathetic communication was crucial.
Bellairs-Walsh et al. (2020) [39]	Qualitative study	The study assessed young individuals' perspectives on the management of suicidal behavior in primary care.	Younger individuals stressed the significance of trust, privacy, and non-judgmental attitudes from GPs.
Jerant et al. (2019) [40]	Qualitative study	The study collected the stakeholders' views regarding planned primary care multimedia suicide prevention tools.	The study disclosed that the stakeholders believed that the multimedia tools could promote disclosure of suicidal thoughts and behaviors, particularly among middle-aged men.
Choo et al. (2019) [41]	Cross-sectional study	The study assessed the clinical assessments of suicide risk and self-reported suicide intent among patient attempters.	The study disclosed that the discrepancies between clinical assessments and patients' self-reported intentions suggest the need for enhanced assessment methods.
Uebelacker et al. (2011) [42]	Cross-sectional study	The study assessed the PHQ-9 depression scale as a key suicide screening tool in primary care settings.	PHQ-9 indicated moderate sensitivity (0.69) and high specificity (0.84) with regard to the identification of suicide risk.
Vannoy and Robins (2011) [43]	Mixed methods analysis	The study analyzed suicide-related discussions between primary care physicians and depressed patients.	The study disclosed that suicide was only discussed in 11% of meetings and that the discussions were increasingly common with female patients.
Saini et al. (2021) [44]	Qualitative study	The study explored the importance of the involvement of patients and the public in health service studies/research.	The study highlighted the positive impact of public involvement on research relevance and implementation.
Crawford et al. (2011) [46]	Randomized controlled trial	The study evaluated the impact of suicide risk screening on primary care patients who had depression.	The study disclosed that screening did not increase distress and might be helpful in identifying individuals at risk of suicide.
Michail et al. (2020) [47]	Commentary	The study discussed various strategies to improve youth suicide prevention within primary care settings.	The study stressed the importance of training and integrated care interventions.
Finnegan et al. (2018) [48]	Implementation study	The study evaluated the various barriers to suicide risk assessment within the primary care settings.	The study identified the significance of improved training and adequate resources for effective suicide risk assessment.
Gordon et al. (2020) [50]	Viewpoint	The study outlined research priorities for suicide prevention within primary care settings.	The study has proposed integrated care models and enhanced data systems.
Graney et al. (2020) [51]	Mixed methods study	The study evaluated suicide risk assessment practices in the UK mental health services.	The study disclosed increased variability in tools utilized and emphasized the importance of standardization.
Miller et al. (2017) [52]	Multisite trial	The study assessed suicide prevention interventions within the emergency departments (ED-SAFE study).	The study disclosed that the various interventions reduced suicide attempts and enhanced patient care outcomes.
Heisel et al. (2010) [53]	Cross-sectional study	The study evaluated depression screening tools used in the detection of suicide ideation in older individuals.	The study disclosed that brief screens were effective in identifying suicidal thoughts.
Binder et al. (2020) [54]	International utility study	The study tested the BITS test used in the detection of adolescent suicidality within primary care settings.	The study has indicated the effectiveness of the test's utility in identifying at-risk youth.
Jerant et al. (2020) [55]	Randomized controlled trial	The study assessed an intervention to promote suicidal thoughts discussions among middle-aged men.	The study disclosed that a customized program increased patient-provider discussions on suicide.
Elzinga et al. (2020) [56]	Qualitative study	The study explored the engagement of primary care professionals in suicide prevention.	The study has identified facilitators and barriers to the implementation of various prevention practices.
Bryan et al. (2023) [57]	Comparative study	The study compared suicide risk screening to depression screening within the primary care context.	The study has disclosed that depression screening was more effective in identifying patients at risk of suicide.
Ford et al. (2021) [59]	Conversation analysis	The study analyzed the discussions about self-harm and suicide in primary care contexts.	The study has underscored the moral and practical intricacies of such conversations.
McCabe et al. (2017) [60]	Qualitative study	The study assessed how healthcare professionals evaluated suicide risk during patient interviews.	The study disclosed that negatively phrased interview questions were hindering accurate disclosure.
Wärdig et al. (2022) [61]	Qualitative study	The study explored nurses' experiences with suicide risk assessment in telephone counseling.	The study has stressed the importance of training and support within telehealth settings.
Wainwright et al. (2020) [63]	Qualitative study	The study involved semi-structured interviews with parents who have been bereaved by suicide in a bid to assess their experiences with primary care support systems.	The study identified themes including the significance of not feeling alone, the various barriers to support access, and the importance of better signposting. It also stressed the vital role of primary care in the provision of support to bereaved parents.
Kim et al. (2021) [64]	Quantitative study using machine learning	The study applied machine learning algorithms to PHQ-9 questionnaire data with the objective of determining the most effective items in screening for suicidal ideation.	The study disclosed that the inclusion of PHQ-9 significantly enhances the accuracy of suicide risk prediction and that the machine learning models showed higher predictive values, signifying their effectiveness in primary care settings.

Risk of Bias Assessment

The assessment of the included studies' quality was conducted using the Appraisal tool for Cross-Sectional Studies (AXIS), which is an important appraisal tool for studies, comprising 20 items [30]. Thus, all the included studies were evaluated by three independent reviewers, and all potential disagreements were resolved through group discussions and consensus. Each included study was scored 1 (yes), 0 (no), or "unclear" for the inapplicable items. Overall, the quality of the included studies was high, as all studies had overall scores that ranged between 16 and 20. Table 2 presents the results of the quality assessment conducted using the AXIS appraisal tool.

**Table 2:** Results of the quality assessment conducted using the AXIS appraisal tool Y: yes, N: no, U: unclear, AXIS: Appraisal tool for Cross-Sectional Studies

Study	Clear aims? (Y/N/U)	Appropriate design? (Y/N/U)	Sample representative? (Y/N/U)	Right sample size? (Y/N/U)	Non-responders? (Y/N/U)	Measures defined? (Y/N/U)	Valid measures? (Y/N/U)	Bias addressed? (Y/N/U)	Ethics? (Y/N/U)	Results clear? (Y/N/U)	Conclusions justified? (Y/N/U)	Overall score
Mesec et al. [31]	Y	Y	Y	Y	N	Y	Y	Y	Y	Y	Y	17
Schou Pedersen et al. [32]	Y	Y	Y	Y	Y	Y	Y	Y	Y	Y	Y	20
Alothman et al. [33]	Y	Y	Y	Y	Y	Y	Y	Y	Y	Y	Y	20
Webb et al. [34]	Y	Y	Y	Y	Y	Y	Y	Y	Y	Y	Y	20
Younes et al. [36]	Y	Y	Y	Y	N	Y	Y	Y	Y	Y	Y	18
Muñoz-Sánchez et al. [37]	Y	Y	Y	Y	N	Y	Y	Y	Y	Y	Y	19
Richards et al. [38]	Y	Y	Y	Y	N	Y	Y	Y	Y	Y	Y	18
Bellairs-Walsh et al. [39]	Y	Y	Y	Y	N	Y	Y	Y	Y	Y	Y	17
Jerant et al. [40]	Y	Y	Y	Y	N	Y	Y	Y	Y	Y	Y	18
Choo et al. [41]	Y	Y	Y	Y	Y	Y	Y	Y	Y	Y	Y	20
Uebelacker et al. [42]	Y	Y	Y	Y	N	Y	Y	Y	Y	Y	Y	18
Vannoy and Robins [43]	Y	Y	Y	Y	N	Y	Y	Y	Y	Y	Y	16
Saini et al. [44]	Y	Y	Y	Y	N	Y	Y	Y	Y	Y	Y	18
Crawford et al. [46]	Y	Y	Y	Y	Y	Y	Y	Y	Y	Y	Y	20
Michail et al. [47]	Y	Y	Y	N	N	Y	Y	Y	Y	Y	Y	16
Finnegan et al. [48]	Y	Y	Y	N	N	Y	Y	Y	Y	Y	Y	16
Gordon et al. [50]	Y	Y	Y	N	N	Y	Y	Y	Y	Y	Y	19
Graney et al. [51]	Y	Y	Y	Y	Y	Y	Y	Y	Y	Y	Y	20
Miller et al. [52]	Y	Y	Y	Y	Y	Y	Y	Y	Y	Y	Y	20
Heisel et al. [53]	Y	Y	Y	Y	N	Y	Y	Y	Y	Y	Y	17
Binder et al. [54]	Y	Y	Y	Y	N	Y	Y	Y	Y	Y	Y	18
Jerant et al. [55]	Y	Y	Y	Y	Y	Y	Y	Y	Y	Y	Y	20
Elzinga et al. [56]	Y	Y	Y	Y	N	Y	Y	Y	Y	Y	Y	17
Bryan et al. [57]	Y	Y	Y	Y	Y	Y	Y	Y	Y	Y	Y	20
Ford et al. [59]	Y	Y	Y	Y	N	Y	Y	Y	Y	Y	Y	19
McCabe et al. [60]	Y	Y	Y	Y	N	Y	Y	Y	Y	Y	Y	18
Wärdig et al. [61]	Y	Y	Y	Y	N	Y	Y	Y	Y	Y	Y	18
Wainwright et al. [63]	Y	Y	Y	Y	N	Y	Y	Y	Y	Y	Y	18
Kim et al. [64]	Y	Y	Y	Y	Y	Y	Y	Y	Y	Y	Y	20

Data Extraction and Synthesis

To effectively extract data from the included studies, we utilized a data extraction form. Data regarding the different study attributes, such as the authors' names, publication year, study sample size, research design, and findings, were collected from all studies. The data was extracted by three reviewers independently, and all potential disagreements were resolved through consensus and discussions among the reviewers. We performed a narrative synthesis structured around key themes (attendance, barriers, screening tools, training, communication, and collaboration). No meta‑analysis was conducted due to heterogeneity in study designs and outcome measures.

Study Findings

Attendance before suicide: Regarding the aspect of attendance at a primary care clinic before suicide commission, the retrospective studies have disclosed that most patients consulted with the primary care clinicians before attempting or committing suicide [31,32]. Studies found that while female patients had more frequent primary care visits, male patients were more likely to die by suicide [33,34]. An additional study also disclosed that, in comparison to the control group, individuals who completed suicide had more primary care physician (PCP) visits within 10 years and no visits during the final month before their deaths [35]. Further, younger persons aged between 18 and 39 years were found to be less liable to seek PCP interventions before suicide attempts compared to older persons aged 40 years and above [36].

Barriers to disclosure: Still, in reviewing the reasons underlying the non-disclosure of suicidal behaviors, three studies have reported different reasons and barriers for the non-disclosure of such behaviors [37-39]. Anxiety surrounding hospitalization and the effects on perceived masculinity concerning the expression of individual vulnerabilities were found to be a major barrier to male patients disclosing their suicidal behaviors [40]. According to Bellairs-Walsh et al., the focus group discussions comprising younger adults aged between 16 and 25 years revealed that the loss of privacy during the revelation of suicidal thoughts was a key barrier to disclosing suicidal behaviors, in addition to the application of labels that include "risk" [39]. An additional study has indicated that the setting and timing of screening questions linked to suicide and self-harm were significant in patient disclosures, alongside the balancing of the perceived disclosure risks, including hospitalization, judgment, and stigma, with the advantage of gaining the necessary support [38]. Still, for primary care clinicians, notable barriers to risk evaluation, screening, and disclosure included a lack of adequate time for assessments and appointments [37].

Screening practices: Concerning screening for suicide risk, it is noteworthy that screening within the PCP entailed the use of shorter evidence-based tools in the identification of at-risk patients requiring additional assessments. This might entail the assessment of the physical condition of the patient, initial suicide attempts, drug and alcohol use, the patient's current mental state, psychological factors, history of mental illness, and determination of extant suicide risk. The reviewed studies have indicated that primary care clinicians did not conduct regular patient screening for suicidal ideation [41,42] and that patients who underwent screening were mostly reported to be low risk after the assessment [43]. This underscores the significance of offering effective screening and treatment of individuals at divergent risk levels [44,45]. It has also been acknowledged that both patients and primary care clinicians should recognize the significance of routine screening for suicidal behaviors and thoughts, and that the employment of such questions does not proffer adverse impacts on the sense of self-worth of the patients [46].

Training and implementation of screening tools: Consequently, several reviewed studies have indicated the significance of increased training of primary care clinicians and staff with regard to the use of suicide risk screening tools, as well as the appropriate interventions, and signposting during the assessment of the risk of suicide in patients [42,45,46,48]. The suicide risk screening tools' validity and significance have been widely and consistently debated [49]. However, a limited number of studies have expressly focused on the impact of the various suicide prevention practices in relation to the long-term patient care outcomes within the primary care contexts [50]. A recent UK study focusing on suicide risk assessment disclosed that there was increased emphasis on the use of suicide risk screening in the identification of individuals at risk compared to the initiation of evidence-based mental health interventions aimed at preventing such outcomes [51]. In this regard, it is noteworthy that suicide screening does not reduce suicide attempts, particularly in instances where effective clinical interventions have not been executed [52]. Although various training interventions and screening tools, including the Geriatric Depression Scale (GDS) [53], Brief Inventory of Thriving (BIT) [54], Men and Providers Preventing Suicide (MAPS) [55], Suicide Prevention Action Networks (SUPRANET) [56], and Patient Health Questionnaire-2 (PHQ-2) [57], have been acknowledged as effective in supporting primary care clinicians in identifying suicide risk, they have to be used together with other interventions, including the use of databases in highlighting the risk factors and training of primary care clinicians, as opposed to being the only intervention. An educational intervention for primary care physicians on depression and suicide prevention increased antidepressant prescriptions but did not reduce suicides [58].

Communication and language factors: Additionally, a number of studies reviewed have acknowledged the significance of the language employed by primary care physicians during interactions with their patients [59,60]. The studies disclosed that the utilization of closed gateway questions, such as directly asking the patients if they had thoughts of harming themselves, resulted in the patients responding that they had no suicidal behaviors and were not suicidal [61]. A comparable study also disclosed that, in certain instances, primary care clinicians inadvertently reinforced the notion of visiting patients not disclosing their suicidal thoughts and behaviors through no-problem-anticipated phrases, such as "you do not feel suicidal, do you?" [62]. Effective communication was perceived as an important aspect in ensuring that the patients and parents bereaved by suicide are comfortable [55,63]. Therefore, asking the patients evidence-based questions, including questions on the "thoughts that you would be better off dead or of hurting yourself in some way," drawn from the Patient Health Questionnaire-9 (PHQ-9), is dependably precise in the screening of persons at risk of suicidality [64].

Inter‑specialty collaboration: Further, regarding the variations observed in suicide risk assessments across the different health services, the studies have disclosed that the evaluation of suicide risk varied significantly between primary care clinicians and other specialties, including mental health departments [45]. However, improved communication between different specialties has also been acknowledged as a major area requiring improvement, and the patients reported improvements in instances where the primary care professionals collaboratively worked as a team [56,65]. For instance, communication through clear and comprehensible language and increased emphasis on using media over text has also been found to be important in communicating with patients within the primary care contexts [55].

Discussion

This systematic review included 15 recent studies that explored how suicide risk factors can be screened and identified within the primary care contexts by the PCPs. The significant role of primary care in the prevention of suicide has been consistently highlighted in various policies, programs, and national strategies. Thus, early risk detection is considered an important aspect of suicide prevention [66,67]. Various recent death registry studies have indicated that approximately 82% of individuals (youths and adults) who completed suicide had initially visited their primary healthcare providers within 12 months before their deaths [66]. Such observations have positioned healthcare visits as potential means for the identification of individuals at high risk of suicidality and subsequently bridging them to effective mental healthcare. Nevertheless, individuals are less liable to discuss their suicidal thoughts in instances where they are not directly asked [66,68-70]. As such, the use of various suicide risk screening tools within the primary care contexts offers clinicians evidence-based questions that they should ask and subsequently enables patients to disclose and discuss their suicidal behaviors and thoughts. According to Mughal et al., suicide screening within the primary care context is a public health intervention with the potential to save lives [70]. This observation has further been supported by various organizations, including The Joint Commission (TJC), the American Academy of Pediatrics (AAP), the American Foundation for Suicide Prevention (AFSP), and the National Action Alliance for Suicide Prevention [71-73]. Nevertheless, a larger proportion of individuals who attempt suicide tend to pass through the primary care systems undetected, given that, in part, many of the medical settings fail to utilize universal and systematic screenings.

Although there have been debates regarding the use of universal screening for suicide within the primary care contexts, there is a universal sentiment that such a type of screening should only be performed in instances where there is a stronger dedication to the provision of treatment and follow-ups [26,74-77]. Although many suicide screening tools are in existence today, there is no single tool that applies to all individuals at high risk for suicide. As such, Stoven et al. recommend that one should not be exclusively reliant on screening tools but also seek to understand patients and their mental health histories [78]. Further, in assessing the different types of existing suicide screening tools, particularly targeted screening and universal screening, it has been noted that, in most nations, including the UK, Australia, Canada, and other European nations, there is moderate evidence indicating recommendation of screening for suicide within primary care contexts [69,78].

Still, available evidence has indicated that suicide screening within the primary care contexts improves care outcomes in instances where it is linked to effective follow-ups and treatments [70,78]. Nonetheless, suicide screening without follow-ups is not recommended, given the insufficient evidence indicating that it is effective in preventing and reducing suicide risk. Rather than universal suicide screening, certain professionals have recommended targeted screening, just as performed in England and Wales [78,79].

The United States Preventive Services Task Force (USPSTF) has recommended universal suicide screening for adults within the primary care context paired with various resources for treatment, diagnostic accuracy, and follow-up [80]. The USPSTF has also recommended universal suicide screening for individuals aged 12 years and above, and this has also been endorsed by the American Academy of Pediatrics [80]. Existing literature further indicates that suicide screening and treatment are vital in preventing suicide [58,81,82].

Regardless of the above observation, a study conducted by the USPSTF in 2014 disclosed that there was inadequate evidence to enable recommendation of or against the use of universal suicide screening within the primary care contexts for adolescents, adults, and elderly persons lacking a mental health diagnosis [80]. Similar conclusions have been reached by a study that focused on suicide prevention strategies [83] and another study that focused on the US Veteran primary care populations [84]. Universal screening has remained an increasingly controversial subject within suicide prevention groups, given the potential for suicidality to take place even in the absence of identifiable risk factors [76]. In this regard, recent studies have disclosed that only 39.5% of adolescents, aged between 10 and 21 years, who are medically hospitalized and screened positively for suicidality, satisfied the depression criteria [74,75].

Nonetheless, suicide screening in high-risk persons is recommended as a set care standard. Within the primary care context, such high-risk persons include individuals with mental health and substance use disorders, as well as individuals on psychotropic medications [75,85,86]. In a cross-sectional study comprising 74 German primary care clinicians, other suicidality predictors, including male sex, depression severity, and physical pain, were added [87]. Some of the recent population-based studies have disclosed rates that are increasing at higher rates among Black, Pacific Islanders, and Asian youths, especially women [88]. Other notable population-based studies have indicated significantly varying rates per subpopulation based on geographic locations [68].

Although the implementation of suicide screening within the primary care contexts has been reported to facilitate early identification of persons at high risk of suicide, there are still challenges in ascertaining timely follow-up and treatment provision. In this light, it has been disclosed that only 32% of individuals who screened positive for suicide were accorded clinical follow-ups within 90 days after hospital visit, indicating the existing gap in care continuity [89,90].

The Henry Ford Health System's "Perfect Depression Care" initiative screens individuals at high risk for suicidality and subsequently categorizes them into severe/acute, high, moderate, and low risk based on factors that include screening, protective factors, and risk factors [91]. This is followed by the program connecting high-risk persons with targeted care based on the identified risk levels [91]. In this regard, Weber et al. [85] and Ramchand et al. [88] have recommended that hospitals should ascertain that patients in high-risk populations are screened.

Additionally, this study's findings have disclosed that the assessment of an individual's possibility of dying through suicide presents a lower positive predictive value and, as a result, must not be the objective of screening for suicide within the primary care contexts [67,81,87]. The focus is on the prevention of suicide within the primary care context. Thus, within the primary care settings, the focus of suicide prevention should be on the recognition and response to the various risk factors, including substance misuse and comorbid physical health diagnoses. The reviewed studies have disclosed that having discussions on suicide and responding to the various suicide risk factors within primary care settings did not increase suicidal behaviors among patients but might have aided in their prevention [80,81,91]. As such, additional trainings are required for primary care staff with regard to the assessment of risks and communication of suicidal behaviors. Presently, several collaborative efforts aimed at standardizing research procedures are ongoing across the globe. Nevertheless, to effectively mitigate suicide and the associated risk factors within primary care contexts, certain strategies and components, including person-centered suicide risk screening, should be included [92,93].

Further, the assessed studies have disclosed that training is a vital primary care component [72,85]. As such, there is a need to allot additional funds for the development of effective learning resources for suicide risk screening, in addition to increasing training within the existing PCP degree programs. The observed lack of consistency with regard to the identification of effective training strategies might indicate that the primary care physicians are at diverse development phases based on the study type being undertaken, funding sources, and training requirements for the organization. Training inconsistencies might also bring about variations in the identification and subsequent treatment of individuals at higher risk of suicide within the primary care contexts [94].

In summary, this study underscores the requirement for primary care physicians and staff to be trained on the identification of suicide risk levels and on how to respond. Notable strategies that might aid in the realization of this include the development of individual-centered care involving the use of validated suicide risk screening assessment to assist in communications on suicide [67,81]. Nonetheless, such strategies are dependent on having increasingly knowledgeable and able primary care physicians and staff adequately trained to routinely discuss suicide with patients presenting various risk factors. Patient-centered and customized strategies should be beneficial to ensure that patients get the necessary and suitable treatment. Collaborative working between primary care clinicians and patients, particularly with regard to service redesign, has additionally been shown to enhance patient care and healthcare outcomes [94]. Patient perspectives should be considered when developing suicide prevention programs, alongside patient engagement in clinical trials [44,87].

## Conclusions

In conclusion, this study has highlighted the crucial role of primary care physicians and clinicians in the prevention of suicide, underscoring the significance of early and timely risk identification and the execution of targeted interventions. While the findings of this systematic review have indicated that most individuals who complete suicide have visited primary care within 12 months prior, available intervention opportunities have regularly been missed as a result of inconsistent suicide risk screening practices. Even as universal suicide risk screening remains widely debated, particularly without any assured follow-up, targeted suicide risk screening, including for individuals with substance and mental health disorders, has been widely supported. This systematic review has underscored the significance of integrating suicide risk screening with timely and well-coordinated care to ascertain efficiency. Notably, no single tool is sufficient; a comprehensive and person-centered intervention that integrates patient history and clinical judgment is important. Support and adequate training of primary care providers have remained inconsistent, indicating the importance of standardized educational initiatives and resource allocations. Eventually, the integration of various validated suicide risk screening tools with collaborative and patient-centered care will significantly improve early identification and improvement of outcomes in suicide prevention within the primary care contexts.
